# Supplementary material for: Modeling the spectrum and determinants of multimorbidity risk among older adults in India
Source: PLoS One. 2025 May 16;20(5):e0323744. doi: 10.1371/journal.pone.0323744 (PMC12083837; doi:10.1371/journal.pone.0323744)
Supplement: S1 Table — (DOCX) [file pone.0323744.s001.docx]

**S1 Table. Morbidity and multimorbidity prevalence among older adults, Longitudinal Ageing Study in India (LASI), wave-1, 2017–2018**

| **Multimorbidity** | **Frequency** | **Weighted Percentage**  ***(N= 59,830)*** |
| --- | --- | --- |
| No Multimorbidity | 33,986 | 56.80 |
| Multimorbidity | 25,844 | 43.20 |
| **Single Morbidities** |  | |
| Musculoskeletal Disorders | 9,771 | 16.33 |
| CVDs | 16,932 | 28.30 |
| Chronic Lung Diseases | 3,956 | 6.61 |
| Eye Disorders | 28,723 | 48.01 |
| Neurological and Psychiatric Disorder | 1,374 | 2.30 |
| Endocrine Diseases | 7,107 | 11.88 |
| Infectious Diseases | 2,114 | 3.53 |
| Cancer | 373 | 0.62 |
| Cholesterol | 1,358 | 2.27 |
| Hearing Disorders | 3,985 | 6.66 |
| Gastrointestinal Conditions | 10,940 | 18.29 |
| Urogenital Diseases | 3,886 | 6.49 |
