# Supplementary material for: Modeling the spectrum and determinants of multimorbidity risk among older adults in India
Source: PLoS One. 2025 May 16;20(5):e0323744. doi: 10.1371/journal.pone.0323744 (PMC12083837; doi:10.1371/journal.pone.0323744)
Supplement: S3 Table — (DOCX) [file pone.0323744.s003.docx]

**S3 Table. Multimorbidity prevalence by exogenous factors among older adults, Longitudinal Ageing Study in India (LASI), wave-1, 2017–2018.**

| **Covariates** | **Multimorbidity (two or more)** | **Frequency**  ***(N= 59,830)*** |
| --- | --- | --- |
| **Age Group** |  |  |
| 45- 49 | 30.64 | 3,470 |
| 50 - 54 | 35.42 | 4,353 |
| 55 - 59 | 44.44 | 2,836 |
| 50 - 64 | 43.99 | 4,038 |
| 65 - 69 | 51.34 | 4,519 |
| 70 - 74 | 54.28 | 3,049 |
| 75 - 79 | 58.1 | 2,394 |
| 80 - 85 | 54.89 | 514 |
| 85 + | 57.04 | 672 |
| **Sex** |  |  |
| Male | 41.63 | 11,416 |
| Female | 44.52 | 14,428 |
| **Residence** |  |  |
| Rural | 38.31 | 16,029 |
| Urban | 54.55 | 9,815 |
| **MPCE quintile** *^a^* |  |  |
| Poorest | 33.46 | 4,220 |
| Poorer | 40.58 | 5,161 |
| Middle | 41.57 | 5,071 |
| Richer | 48.38 | 5,692 |
| Richest | 54.09 | 5,701 |
| **Highest level of Schooling** |  |  |
| No Schooling | 37.47 | 11,352 |
| < 5 Years | 47.48 | 3,172 |
| 5 - 9 Years | 46.75 | 5,793 |
| 10 + Years | 52.81 | 5,527 |
| **Religion** |  |  |
| Hindu | 42.13 | 20,799 |
| Muslim | 48.42 | 3,194 |
| Christian | 41.15 | 726 |
| Other | 53.6 | 1,125 |
| **Caste Category** |  |  |
| ST | 25.46 | 1,314 |
| SC | 40.19 | 4,679 |
| OBC | 42.74 | 11,649 |
| Other | 52.01 | 8,202 |
| **Working Status** |  |  |
| Never worked | 49.2 | 7,675 |
| Currently working | 35.27 | 10,619 |
| Currently not working | 53.48 | 7,526 |
| **Current Marital Status** |  |  |
| Currently married | 41.24 | 18,242 |
| Widowed | 50.54 | 7,028 |
| Divorced/separated/other | 34.03 | 574 |
| **Region** |  |  |
| North | 47.68 | 3,610 |
| Central | 29.93 | 3,749 |
| East | 43.48 | 6,194 |
| Northeast | 37.26 | 772 |
| West | 50.51 | 4,789 |
| South | 48.31 | 6,730 |
| **Alcohol Consumption** |  |  |
| Lifetime abstainer | 43.95 | 22,011 |
| Infrequent non-heavy drinker | 47.29 | 1,800 |
| Frequent non-heavy drinker | 36.52 | 536 |
| Heavy episodic drinker | 29.50 | 219 |
| **Tabacco Consumption** |  |  |
| Lifetime abstainer | 45.24 | 16,866 |
| Smokes tobacco | 40.74 | 3,471 |
| Smokeless tobacco | 39.05 | 4,764 |
| Both | 40.69 | 743 |
| **Childhood Health** |  |  |
| Very good | 43.74 | 12,736 |
| Good | 42.26 | 9,767 |
| Fair | 43.05 | 2,842 |
| Poor | 49.54 | 440 |
| Very Poor | 80.96 | 60 |
| **BMI** ^b^ |  |  |
| Underweight | 32.47 | 3,990 |
| Normal | 40.55 | 12,698 |
| Overweight | 53.79 | 6,534 |
| Obese | 64.25 | 2,621 |
| **Physical Activity** |  |  |
| Everyday | 34.68 | 5,296 |
| Weekly | 36.34 | 2,291 |
| Casual | 47.73 | 18,219 |
| Footnote:  *a - Wealth index*  *b - Body mass index, Underweight (BMI≤ 18.4 kg/m2), Normal (18.5 kg/m2 ≤ BMI ≤ 24.9 kg/m2), Overweight (25.0 kg/m2 ≤ BMI ≤ 29.9 kg/m2), Obese (BMI ≥ 30 kg/m2)* | | |
