# Supplementary material for: Modeling the spectrum and determinants of multimorbidity risk among older adults in India
Source: PLoS One. 2025 May 16;20(5):e0323744. doi: 10.1371/journal.pone.0323744 (PMC12083837; doi:10.1371/journal.pone.0323744)
Supplement: S4 Table — (DOCX) [file pone.0323744.s004.docx]

**S4 Table. VIMP from RF analysis of multimorbidity and no multimorbidity, Longitudinal Ageing Study in India (LASI), wave-1, 2017–2018.**

| **Covariates** | **Multimorbidity** | **No Multimorbidity** |
| --- | --- | --- |
| BMI | 0.0581 | -0.0196 |
| Sex | 0.0467 | -0.0059 |
| Highest level of Schooling | 0.0241 | 0.0121 |
| Current Marital Status | 0.0240 | 0.0681 |
| Alcohol Consumption | 0.0160 | 0.0210 |
| Caste Category | 0.0115 | 0.0052 |
| Religion | 0.0114 | 0.0063 |
| Childhood Health | 0.0108 | 0.0123 |
| MPCE quintile | 0.0081 | -0.0050 |
| Tobacco Consumption | 0.0073 | 0.0054 |
| Region | 0.0062 | 0.0014 |
| Physical Activity | 0.0051 | 0.0222 |
| Residence | 0.0015 | -0.0053 |
| Working Status | -0.0072 | 0.1147 |
