# Supplementary material for: Modeling the spectrum and determinants of multimorbidity risk among older adults in India
Source: PLoS One. 2025 May 16;20(5):e0323744. doi: 10.1371/journal.pone.0323744 (PMC12083837; doi:10.1371/journal.pone.0323744)
Supplement: Attachment — Submitted filename: Response to Reviewers.docx [file pone.0323744.s006.docx]

To, The Editor, Reviewers,

PLOS ONE

Date: January 16, 2025

Subject: Addressing the Editor and Reviewers comments for PONE-D-24-52393

Dear Editor, Reviewers

We sincerely appreciate your valuable feedback and the time you took to review our work. Your comments were highly insightful and significantly contributed to enhancing the quality of our paper. We have made every effort to address your concerns thoroughly and thoughtfully.

Kindly help us with your further concerns (if any) to improvise our paper. Below, we have provided detailed responses to each of your comments, along with the actions taken to address them. For clarity, our responses have been highlighted in brown.

Regards,

Authors of PONE-D-24-52393

PONE-D-24-52393

Modeling the Spectrum and Determinants of Multimorbidity Risk among older adults in India

PLOS ONE

Dear Dr. Kumar,

Thank you for submitting your manuscript to PLOS ONE. After careful consideration, we feel that it has merit but does not fully meet PLOS ONE’s publication criteria as it currently stands. Therefore, we invite you to submit a revised version of the manuscript that addresses the points raised during the review process.

==============================

Please address the comments by the reviewers, and address the citation and typographical errors.
==============================

Please submit your revised manuscript by Feb 24 2025 11:59PM. If you will need more time than this to complete your revisions, please reply to this message or contact the journal office at [plosone@plos.org](mailto:plosone@plos.org). When you're ready to submit your revision, log on to https://www.editorialmanager.com/pone/ and select the 'Submissions Needing Revision' folder to locate your manuscript file.

Please include the following items when submitting your revised manuscript:

- A rebuttal letter that responds to each point raised by the academic editor and reviewer(s). You should upload this letter as a separate file labeled 'Response to Reviewers'.

Included

- A marked-up copy of your manuscript that highlights changes made to the original version. You should upload this as a separate file labeled 'Revised Manuscript with Track Changes'.

Included

- An unmarked version of your revised paper without tracked changes. You should upload this as a separate file labeled 'Manuscript'.

Included

If you would like to make changes to your financial disclosure, please include your updated statement in your cover letter. Guidelines for resubmitting your figure files are available below the reviewer comments at the end of this letter.

If applicable, we recommend that you deposit your laboratory protocols in protocols.io to enhance the reproducibility of your results. Protocols.io assigns your protocol its own identifier (DOI) so that it can be cited independently in the future. For instructions see: <https://journals.plos.org/plosone/s/submission-guidelines#loc-laboratory-protocols>. Additionally, PLOS ONE offers an option for publishing peer-reviewed Lab Protocol articles, which describe protocols hosted on protocols.io. Read more information on sharing protocols at <https://plos.org/protocols?utm_medium=editorial-email&utm_source=authorletters&utm_campaign=protocols>.

We look forward to receiving your revised manuscript.

Kind regards,

Emmanuel Kokori, M.D
Academic Editor
PLOS ONE

Journal Requirements:

When submitting your revision, we need you to address these additional requirements.

1. Please ensure that your manuscript meets PLOS ONE's style requirements, including those for file naming. The PLOS ONE style templates can be found at

https://journals.plos.org/plosone/s/file?id=wjVg/PLOSOne_formatting_sample_main_body.pdf and

<https://journals.plos.org/plosone/s/file?id=ba62/PLOSOne_formatting_sample_title_authors_affiliations.pdf>

Addressed

2. PLOS requires an ORCID iD for the corresponding author in Editorial Manager on papers submitted after December 6th, 2016. Please ensure that you have an ORCID iD and that it is validated in Editorial Manager. To do this, go to ‘Update my Information’ (in the upper left-hand corner of the main menu), and click on the Fetch/Validate link next to the ORCID field. This will take you to the ORCID site and allow you to create a new iD or authenticate a pre-existing iD in Editorial Manager.

Included

3. Please include captions for your Supporting Information files at the end of your manuscript, and update any in-text citations to match accordingly. Please see our Supporting Information guidelines for more information: <http://journals.plos.org/plosone/s/supporting-information>.

Included

[Note: HTML markup is below. Please do not edit.]

Reviewers' comments:

Reviewer's Responses to Questions

**Comments to the Author**

1. Is the manuscript technically sound, and do the data support the conclusions?

The manuscript must describe a technically sound piece of scientific research with data that supports the conclusions. Experiments must have been conducted rigorously, with appropriate controls, replication, and sample sizes. The conclusions must be drawn appropriately based on the data presented.

Reviewer #1: Partly

Reviewer #2: Partly

2. Has the statistical analysis been performed appropriately and rigorously?

Reviewer #1: I Don't Know

Reviewer #2: No

3. Have the authors made all data underlying the findings in their manuscript fully available?

The [PLOS Data policy](http://www.plosone.org/static/policies.action#sharing) requires authors to make all data underlying the findings described in their manuscript fully available without restriction, with rare exception (please refer to the Data Availability Statement in the manuscript PDF file). The data should be provided as part of the manuscript or its supporting information, or deposited to a public repository. For example, in addition to summary statistics, the data points behind means, medians and variance measures should be available. If there are restrictions on publicly sharing data—e.g. participant privacy or use of data from a third party—those must be specified.

Reviewer #1: Yes

Reviewer #2: Yes

4. Is the manuscript presented in an intelligible fashion and written in standard English?

PLOS ONE does not copyedit accepted manuscripts, so the language in submitted articles must be clear, correct, and unambiguous. Any typographical or grammatical errors should be corrected at revision, so please note any specific errors here.

Reviewer #1: Yes

Reviewer #2: Yes

5. Review Comments to the Author

Please use the space provided to explain your answers to the questions above. You may also include additional comments for the author, including concerns about dual publication, research ethics, or publication ethics. (Please upload your review as an attachment if it exceeds 20,000 characters)

**Reviewer #1:** The manuscript presents an analysis of data from the Longitudinal Ageing Study in India (LASI), around the issue of multimorbidity (MM) and its determinants. They conclude that the prevalence of MM is 43.20%, with eye disorder as the main group of diseases. They conclude also, based on a Random Forest analysis, that obesity, higher levels of education, work and poor childhood health are the main determinants of MM in its population.
The manuscript adresses a relevant issue (MM and health transition in Low and Middle Income (LMIC) countries) supported by data from a national survey. But there are some methodological problems that should be adressed before publication:

**Response**: Thank you for your comments and for recognizing the relevance of our study on multimorbidity (MM) and its determinants in the context of the health transition in LMICs. We appreciate your feedback and have carefully considered your suggestions. In response to the methodological concerns, we have revised the manuscript and made the necessary adjustments. These changes address the specific issues raised and aim to improve the clarity, rigor, and comprehensiveness of our analysis.

**Comment:** The main problem relates to the definition of MM used by the authors. In the bibliography MM is defined as the presence of two or more Long Term Conditions in a patient, specifying the LTC included as individual diseases with a defined impact on the quality of life and survival of the patient (so, with a criteria to identify the disease to be included). In the manuscript the authors use categories of diseases (not individual conditions) and do not specify criteria. Even more so, they refer to diseases when it seems that they use categories of diseases.

- As a result, the most prevalent conditions in the study are Eye dirorders, a category of diseases taht includes such conditions as myopia or refractive disorders that are prevalent and rarely included in MM studies, and not related to other relevant LTC conditions.

**Response**: First, in our study, we defined multimorbidity (MM) in line with the WHO's definition as the "coexistence of two or more chronic conditions in the same individual." This definition does not strictly require the conditions to be classified as long-term or morbidities. Additionally, we acknowledge that our approach categorizes diseases rather than listing individual conditions, aligning with the broader scope of our analysis. Now, relevant references have been provided below for further clarification.

<https://iris.who.int/bitstream/handle/10665/252275/9789241511650-eng.pdf?sequence=1>

<https://www.nature.com/articles/s41572-022-00376-4>

Second, thank you for pointing out the categories of diseases. We have now addressed this point in the "Morbidities" section of the Methods, where we provide a detailed explanation of how morbidities or chronic conditions were categorized. This categorization was based on information from the self-reported and diagnoses available in the LASI dataset, which were documented using participants' responses to specific survey questions such that “Has any health professional ever diagnosed you with the following chronic conditions or diseases?” and “In the past 2 years, have you had any of the following diseases?”

**Comment:** These inconsistencies in the definition of MM lead to contraintuitive results in the study of determinants, as the conslusion that a better education or currently working are related to worst health.

**Response**: We have revised the manuscript and maintained the consistency of MM. Furthermore, the categorization of the morbidities or chronic conditions and detailing the description of constructing the outcome variable (MM) are included and explained in the “Variables Definitions” under the methods section.

Further, we acknowledge that the conclusion suggesting a higher risk of MM among individuals with better education and those currently working may appear counterintuitive. However, this finding aligns with evidence from prior studies, which have similarly reported such patterns. Additionally, our analysis corroborates this trend, as shown in the cross-tabulations of MM with the highest level of schooling and working status variables. Detailed prevalence data stratified by these exogenous factors are presented in S3 Table for further context and clarity.

**Comment:** Another problem relates to obesity, here analyzed as a determinant of health and in other studies considered as a chronic condition on his own. The real variable under study is Body Mass Index (BMI), but the authors refer in the manuscript as obesity.

**Response**: Yes, obesity is often considered a chronic condition in many studies. However, in our study, we examine Body Mass Index (BMI) as a determinant of health, specifically in relation to the occurrence of multimorbidity (MM). BMI has been categorized into four groups: underweight (BMI ≤ 18.4 kg/m²), normal (18.5–24.9 kg/m²), overweight (25.0–29.9 kg/m²), and obese (BMI ≥ 30 kg/m²). Our findings indicate that individuals with a BMI of 30 kg/m² or higher are significantly more likely to experience multimorbidity.

Other major comments:
**Comment:** The study should specify (in supplementary material or in a referenced study, for example) the operative definitions of the variables included in the study or the original survey

**Response**: Thank you for your valuable feedback, we have carefully reviewed and addressed the issue. Detailed descriptions of all the exogenous factors included in the study are now provided in S2 Table of supplementary for clarity and reference.

**Comment:** The Outcome variable is MM, but in some cases it is considered as a binary variable (YES/NO) and in other cases as MM counts (with the problema that it does not use couynts of diseases). This should be specified.

**Response**: In our study, multimorbidity (MM) is analyzed both as a binary variable (YES/NO) and as MM counts, depending on the specific research objective. The binary approach allows us to examine the overall association between multimorbidity and various exogenous factors, such as age, socioeconomic status, and lifestyle, providing a clear distinction between individuals with and without multimorbidity. On the other hand, using MM counts enables us to capture the gradient of disease burden by analyzing the number of coexisting morbidities (typically two or more) and their relationship with exogenous factors. This approach provides deeper insights into how an increasing number of conditions affects the influence of key determinants.

**Comment:** Thouh I am not a statistician, it seems that the results about the association among the different exogenous factors and the outcome variable are only adjusted by age group, not by other variables that can confound the result. For example, association between education and MM can be biased by the BMI or the working status. The authors should present adjusted results, al least by the relevant variables.

**Response**: Thank you for your comment on the adjustment of results by potential confounding variables. We appreciate your concern and agree that adjustments play a critical role in addressing confounding factors in statistical analysis. In our study, we chose to adjust for age groups as a primary stratifying variable due to its central role in multimorbidity research and its strong correlation with both the exposure variables and the outcome. Our primary objective was to capture the risk of multimorbidity across socioeconomic and demographic factors in a straightforward manner, focusing on the unadjusted relationships that might otherwise be obscured by over-adjustment in traditional statistical methods. While it is true that variables like BMI and working status can influence the association between education and multimorbidity, we intended to examine the broad patterns of risk rather than isolate specific causal pathways. We hope this explanation clarifies our methodological choices and rationale.

**Comment:** The definition of some variables (as Tobacco consumption) is not clear. Which category correspond to a person that has smoked for 10 years but does not currently smoke? The study uses prevalence data for all the variables, but does not account for the porevious risk factors.

**Response**: Thank you for your observation regarding the clarity of variable definitions, particularly tobacco consumption. The subcategories of tobacco consumption (Lifetime abstainer, Smokes tobacco, Smokeless tobacco, Both) are based on the respondent’s current usage status. During the interview, participants were specifically asked, “Do you currently consume any Smokes tobacco, Smokeless tobacco, or Both?” Thus, the classification reflects their current tobacco use at the time of the survey.

Other comments:
**Comment:** The authors should introduce the complete definition the first time they use an acronym: CD, NCD, UT, CVD, COPD, BMI, MPCE,SC, OBC, ST…

**Response**: We have revised the acronyms in Table 1 by including their complete definitions in the footnotes to ensure clarity for the readers. Additionally, detailed descriptions of all the exogenous factors referenced in the study have been provided in S2 Table.

**Comment:** Many of the bibliographical references are incomplete or erroneous:
o The PMC references are usually incomplete (ex: /pmc/articles/PMC9832245/ in ref. 45): Ref 1, 4, 5, 7, 10, 12, 13, 14, 17, 27, 30, 45, 48, 49, 52, 54, 55, 58, 59, 60
o References 11, 29, 32, 36 are erroneous (authors should check them)
o Reference 29 is incomplete
o Reference 34 explains the use of Random survival forests analysis in survival studies. As this is not a survival analysis, it would be better to use another reference to Random Forest analysis in descriptive studies.

**Response**: Thank you for your insightful feedback regarding the bibliographical references. We have thoroughly reviewed and addressed the issues you identified:

1. Incomplete PMC References: All PMC references (e.g., /pmc/articles/PMC9832245/) have been updated with complete citations for References 1, 4, 5, 7, 10, 12, 13, 14, 17, 27, 30, 45, 48, 49, 52, 54, 55, 58, 59, and 60.
2. Erroneous References: References 11, 29, 32, and 36 have been carefully examined and corrected to ensure accuracy.
3. Reference 29: This reference was incomplete and has now been fully updated.
4. Reference 34: The previous citation, which pertained to Random Survival Forests analysis, has been replaced with a more relevant reference on Random Forest analysis for descriptive studies, reflecting the methodology used in our work.

We appreciate your detailed observations, which have helped us improve the manuscript’s accuracy and clarity.

**Comment:** Some of the comments on the Results section should be redirected to the Discussion section:
Poor childhood health is telltale signs of early origins of morbidity that have deep down association with high risk of multimorbidity. Morbidity, disability, and deformations played a vital role for developing long-term multimorbidity

This highlights a transition of the risk of complex multimorbidity from obese to normal BMI individuals in the oldest of old age groups, indicating the critical importance of addressing sex-specific and age-related factors in understanding and mitigating multimorbidity risk.

**Response**: Thank you for the suggestion. We have incorporated these insights and discussed the findings in the Discussion section (6^th^ and 7^th^ paragraph).

**Reviewer #2:** It was a pleasure to read this manuscript. However, I have several suggestions and questions for the authors:
**Response**: Thank you for your encouraging comments and suggestions. We have carefully revised the manuscript and updated it accordingly.

**Comment:** The manuscript lacks proper citations in multiple sentences, particularly in the latter part of the introduction.
**Response**: Thank you for the observation. We have addressed the issue and added the necessary citations to the relevant sentences in the latter part of the introduction.

**Comment:** My understanding is that the LASI (Wave 1) survey is not longitudinal on its own; it becomes longitudinal only when subsequent waves (e.g., Wave 2) are included.
The authors have not explained how the aging study incorporates morbidity and multimorbidity. Were specific criteria used to define morbidity conditions, or were these based solely on self-reported data from the participants?

**Response**: Thank you for your observation regarding the nature of the LASI (Wave 1) dataset and its classification as longitudinal only with subsequent waves. We agree with your understanding and acknowledge that Wave 1, on its own, serves as the baseline for longitudinal analysis in future iterations when additional waves are included.

Regarding your question on how the LASI study incorporates morbidity and multimorbidity, we have now addressed this comprehensively in the "Morbidities" section of the Methods. Specifically, the categorization of morbidities or chronic conditions was based on self-reported data and available diagnoses within the LASI dataset. Participants were asked direct survey questions such as, “Has any health professional ever diagnosed you with the following chronic conditions or diseases?” and “In the past 2 years, have you had any of the following diseases?” These responses provided the basis for identifying and categorizing various chronic conditions, which were then used to define multimorbidity. This approach aligns with existing literature and ensures consistency in how multimorbidity is operationalized in population health studies.

**Comment:** The manuscript does not adequately justify why the Random Forest method was chosen over previously used methods (e.g., Sinha et al., 2022: https://doi.org/10.3390/ijerph19159091). A comparison of these methods would strengthen the argument.

**Response**: We selected the Random Forest (RF) method for this study due to its unique strengths in handling complex, non-linear relationships and interactions among variables, which are often present in multimorbidity studies. The RF model's ability to manage high-dimensional data without imposing strict assumptions about variable distributions made it particularly suitable for exploring the associations between multimorbidity and a wide range of socioeconomic and demographic factors. While we recognize that other methods, such as those used in Sinha et al. (2022), have their merits, our choice of the RF method was guided by its robustness in identifying important predictors and its capacity to provide interpretable insights through variable importance measures and conditional plots. These features align with our study’s objective of assessing risk patterns and identifying key contributors to multimorbidity. We acknowledge that a comparison of the RF method with previously employed techniques could add depth to our methodological discussion. While such a comparison was beyond the scope of the current analysis, we appreciate the suggestion

**Comment:** The process for categorizing the 12 major comorbidities is unclear. Did the authors use specific criteria for this categorization?

**Response**: Thank you for the observation. We have addressed this point by explaining in the “Variables Definitions” section of methods that how we categorized the morbidities or chronic conditions and detailing the process of constructing the outcome variable.

**Comment:** In the Statistical Analysis section, the authors state, "The approach of prior studies in India for multimorbidity risk estimation was built on odds ratio or relative risk models, which were not suitable for understanding the non-linear relationship for background characteristics (34)." However, the article introduces only the Random Survival Forest (RSF) method. More discussion and evidence are needed to substantiate this conclusion.

**Response**: We revised the reference to include a more appropriate study and provided additional evidence to further substantiate the conclusion regarding the limitations of traditional models and the advantages of using the Random Survival (RF) method for understanding non-linear relationships in multimorbidity risk estimation.

**Comment:** One significant concern is the lack of discussion about ethical considerations, especially since secondary data from the LASI survey is being used. Additionally, it should be noted that this is not an original research study.

Response: We have addressed this point by adding a new subsection titled "Data Availability and Ethical Considerations" in the Methods section to provide a more detailed discussion on the ethical considerations of the study.

**Comment:** The manuscript introduces the Gini index without citing its source or explaining its application in the analysis.
**Response:** We cited the paper on Gini index in the statistical analysis and application of it is added as “is used to measure the heterogeneity of a dataset at a node. It helps determine the best feature and threshold for splitting a node by minimizing impurity, thereby creating more homogeneous child nodes “

**Comment:** The results presented in Table 3 are not adequately discussed in the manuscript. Including a detailed explanation of these outcomes would enhance the clarity and completeness of the analysis.

**Response**: This comment has been extremely insightful. In the section “3.3 Risk of multimorbidity by leading factors” we have now explained and discussed the VIMP and leading exogenous risk factors for multimorbidity susceptibility.
