# Supplementary material for: Modeling the spectrum and determinants of multimorbidity risk among older adults in India
Source: PLoS One. 2025 May 16;20(5):e0323744. doi: 10.1371/journal.pone.0323744 (PMC12083837; doi:10.1371/journal.pone.0323744)
Supplement: Attachment — Submitted filename: Response_to_Reviewers_auresp_2.docx [file pone.0323744.s007.docx]

To, The Editor, Reviewers,

PLOS ONE

Date: February 19, 2025

Subject: Addressing the Editor and Reviewers comments for PONE-D-24-52393R1

Dear Editor, Reviewers

We sincerely appreciate your valuable feedback and the time you took to review our work. Your comments were highly insightful and significantly contributed to enhancing the quality of our paper. We have made every effort to address your concerns thoroughly and thoughtfully.

Kindly help us with your further concerns (if any) to improvise our paper. Below, we have provided detailed responses to each of your comments, along with the actions taken to address them. For clarity, our responses have been highlighted in **brown.**

Regards,

Authors of PONE-D-24-52393R1

PONE-D-24-52393R1

Modeling the Spectrum and Determinants of Multimorbidity Risk among older adults in India

PLOS ONE

Dear Dr. Kumar,

Thank you for submitting your manuscript to PLOS ONE. After careful consideration, we feel that it has merit but does not fully meet PLOS ONE’s publication criteria as it currently stands. Therefore, we invite you to submit a revised version of the manuscript that addresses the points raised during the review process.

==============================
Please address the comments by reviewer 1.
==============================

Please submit your revised manuscript by Apr 04 2025 11:59PM. If you will need more time than this to complete your revisions, please reply to this message or contact the journal office at [plosone@plos.org](mailto:plosone@plos.org). When you're ready to submit your revision, log on to https://www.editorialmanager.com/pone/ and select the 'Submissions Needing Revision' folder to locate your manuscript file.

Please include the following items when submitting your revised manuscript:

- A rebuttal letter that responds to each point raised by the academic editor and reviewer(s). You should upload this letter as a separate file labeled 'Response to Reviewers'.

Included

- A marked-up copy of your manuscript that highlights changes made to the original version. You should upload this as a separate file labeled 'Revised Manuscript with Track Changes'.

Included

- An unmarked version of your revised paper without tracked changes. You should upload this as a separate file labeled 'Manuscript'.

Included

If you would like to make changes to your financial disclosure, please include your updated statement in your cover letter. Guidelines for resubmitting your figure files are available below the reviewer comments at the end of this letter.

If applicable, we recommend that you deposit your laboratory protocols in protocols.io to enhance the reproducibility of your results. Protocols.io assigns your protocol its own identifier (DOI) so that it can be cited independently in the future. For instructions see: <https://journals.plos.org/plosone/s/submission-guidelines#loc-laboratory-protocols>. Additionally, PLOS ONE offers an option for publishing peer-reviewed Lab Protocol articles, which describe protocols hosted on protocols.io. Read more information on sharing protocols at <https://plos.org/protocols?utm_medium=editorial-email&utm_source=authorletters&utm_campaign=protocols>.

We look forward to receiving your revised manuscript.

Kind regards,

Emmanuel Kokori, M.D
Academic Editor
PLOS ONE

Additional Editor Comments:

Based on the reviewer’s concerns, a revision is required to improve the manuscript’s clarity and methodological rigor. Please address the following:

Thank you for your valuable comments and suggestions. We sincerely appreciate your thoughtful feedback and have made every effort to incorporate all the suggested revisions into the manuscript to enhance its clarity, rigor, and overall quality of our paper.

1. Clarify the Definition of Multimorbidity (MM): Ensure consistency with established definitions in the literature. If using disease categories instead of specific conditions, provide strong justification and acknowledge limitations.

**Response**: We revised the definition of MM as “two or more coexisting conditions in an individual” in the introduction and discussion section, and added the revised reference for this definition as well.

2. Refine Categorization of Conditions: Clearly outline how conditions were selected and classified in the LASI dataset. Consider aligning more closely with standard MM definitions or discussing the implications of your approach.

**Response**: Thank you for your observation. We did not independently categorize the conditions. Instead, the categorization was based on a well-structured criterion, which is detailed in the morbidities section of the manuscript.

3. Strengthen the Risk Factor Analysis: Adjust for potential confounders, especially in the analysis of education and socioeconomic status. Provide a clearer rationale for findings that may appear counterintuitive.

**Response**: Thank you for your comment on the adjustment of results by potential confounding variables. We appreciate your concern and agree that adjustments play a critical role in addressing confounding factors in statistical analysis. In our study, we chose to adjust for age groups as a primary stratifying variable due to its central role in multimorbidity research and its strong correlation with both the exposure variables and the outcome. Our primary objective was to capture the risk of multimorbidity across socioeconomic and demographic factors in a straightforward manner, focusing on the unadjusted relationships that might otherwise be obscured by over-adjustment in traditional statistical methods

Moreover, we now acknowledge the limitation that the risk estimates of MM are not adjusted in the limitation sections of the manuscript.

4. Revise the Discussion: Contextualize findings in light of MM’s revised definition and adjusted analysis. Acknowledge limitations and ensure alignment with cited references.

**Response**: We revise the definition of multimorbidity and updated reference. We also acknowledge the limitation that the diseases are chronic in “complex multimorbidity” in our study. Although, it presents a paradox in our study context—whether complex multimorbidity includes chronic conditions or not. Since, we also considered chronic conditions in our study.

Please submit a detailed response outlining changes made. We look forward to your revised manuscript.

[Note: HTML markup is below. Please do not edit.]

Reviewers' comments:

Reviewer's Responses to Questions

**Comments to the Author**

1. If the authors have adequately addressed your comments raised in a previous round of review and you feel that this manuscript is now acceptable for publication, you may indicate that here to bypass the “Comments to the Author” section, enter your conflict-of-interest statement in the “Confidential to Editor” section, and submit your "Accept" recommendation.

Reviewer #1: (No Response)

Reviewer #2: All comments have been addressed

2. Is the manuscript technically sound, and do the data support the conclusions?

The manuscript must describe a technically sound piece of scientific research with data that supports the conclusions. Experiments must have been conducted rigorously, with appropriate controls, replication, and sample sizes. The conclusions must be drawn appropriately based on the data presented.

Reviewer #1: Partly

Reviewer #2: Yes

3. Has the statistical analysis been performed appropriately and rigorously?

Reviewer #1: I Don't Know

Reviewer #2: Yes

4. Have the authors made all data underlying the findings in their manuscript fully available?

The [PLOS Data policy](http://www.plosone.org/static/policies.action#sharing) requires authors to make all data underlying the findings described in their manuscript fully available without restriction, with rare exception (please refer to the Data Availability Statement in the manuscript PDF file). The data should be provided as part of the manuscript or its supporting information, or deposited to a public repository. For example, in addition to summary statistics, the data points behind means, medians and variance measures should be available. If there are restrictions on publicly sharing data—e.g. participant privacy or use of data from a third party—those must be specified.

Reviewer #1: Yes

Reviewer #2: Yes

5. Is the manuscript presented in an intelligible fashion and written in standard English?

PLOS ONE does not copyedit accepted manuscripts, so the language in submitted articles must be clear, correct, and unambiguous. Any typographical or grammatical errors should be corrected at revision, so please note any specific errors here.

Reviewer #1: Yes

Reviewer #2: Yes

6. Review Comments to the Author

Please use the space provided to explain your answers to the questions above. You may also include additional comments for the author, including concerns about dual publication, research ethics, or publication ethics. (Please upload your review as an attachment if it exceeds 20,000 characters)

**Reviewer #1:** The authors use in this study a definition of Multimorbidity that is not comparable to the definitions included in the bibliographical references of the manuscript. They use different definitions of MM counts or complex MM based on the assumption that a category of diseases or health problems is equivalent to a chronic condition. By doing so they misinterpret the concept of Multimorbidity as it is usually conceptualised in the literature. The references provided by the authors in their answer allign with the definition of MM as the coexistence of two or more Long Term Conditions from a prespecified list of specific diseases.

**Response**: Thank you for the observation. We have revised the definition of multimorbidity in our study to "two or more coexisting conditions in an individual," which does not necessarily require these conditions to be chronic. This revision aligns with the broader conceptualization of multimorbidity in the literature and accounts for the lack of a universally accepted definition. Our approach, which classifies diseases into categories, is a valid and contextually relevant adaptation, particularly in the Indian healthcare setting.

Finally, we acknowledge the limitation of complex multimorbidity in that the diseases are chronic. It presents a paradox in our study context—whether complex multimorbidity includes chronic conditions or not. Since, we also considered chronic conditions in our study.

**Comment**: Moreover, the analysis of risk factors for MM susceptibility concludes that major levles of schooling or better socioeconomic conditions are risk factors for poor health (as throughout the study multimorbidity is compared to poor health). This controversial conclussion is justified by the lack of adjustments in the analysis and the erroneous conceptualization of MM in the study.

**Response**: Thank you for your comment. These findings align with previous studies conducted in the Indian context. Moreover, the prevalence of MM is higher among individuals with higher education levels and better socioeconomic status, with 54% among the richest quintile compared to 33.46% in the poorest quintile. A similar pattern is observed in education levels, where the prevalence of MM is 52.81% among individuals with 10 or more years of schooling, compared to 37.47% among those with no formal education. Moreover, poor health is it the implication of MM instead to poor health as MM.

Finally, we now acknowledge the limitation that the risk estimates of MM are not adjusted in the limitation sections of the manuscript.

**Reviewer #2:** All comments were well addressed by the authors. I appreciate the response from the authors. It's in a stage of publication.

**Response**: Thank you for your encouraging comments and suggestions. Comments helped us to improve the quality of the manuscript.
